# microRNAs as Biomarkers and Therapeutic Targets in Rheumatoid Arthritis

**DOI:** 10.3390/ijms26209950

**Published:** 2025-10-13

**Authors:** Filip Machaj, Magdalena Chmielewska-Jeznach, Anna Koryszewska-Bagińska, Damian Malinowski, Andrzej Pawlik, Gabriela Olędzka

**Affiliations:** 1 Department of Medical Biology, Medical University of Warsaw, ul. Litewska 14/16, 00-575 Warsaw, Poland; filip.machaj@wum.edu.pl (F.M.); magdalena.chmielewska-jeznach@wum.edu.pl (M.C.-J.); anna.koryszewska-baginska@wum.edu.pl (A.K.-B.); 2 Department of Pharmacokinetics and Therapeutic Drug Monitoring, Pomeranian Medical University, 70-111 Szczecin, Poland; damian.malinowski@pum.edu.pl; 3 Department of Physiology, Pomeranian Medical University in Szczecin, 70-111 Szczecin, Poland; pawand@poczta.onet.pl

**Keywords:** rheumatoid arthritis, microRNA, synoviocytes, therapy

## Abstract

Rheumatoid arthritis (RA) is a prevalent autoimmune disease characterized by chronic joint inflammation. Its pathophysiology involves complex interactions among immune cells, leading to joint damage, primarily in the synovial membrane. MicroRNAs (miRs), single-stranded non-coding RNAs, play a critical role in regulating pathways affecting RA progression, particularly in fibroblast-like synoviocytes and peripheral blood mononuclear cells. Key pathways influenced by miRs include NF-κB, apoptosis, PI3K/AKT signaling, and cytokine production. Dysregulated miRs impact cell proliferation, survival, and inflammatory responses. This review explores not only the role of miRs in RA pathogenesis, but also highlights their potential as biomarkers for early detection and severity prediction. Moreover, therapeutic approaches targeting miRs, including mimics and inhibitors, show promise in animal models, with methods like intra-articular administration being favored due to better efficacy and reduced side effects. While early studies highlight potential pathways for RA treatment, challenges remain in translating these findings into safe and effective clinical therapies.

## 1. Introductions

Rheumatoid arthritis (RA) is a chronic autoimmune disease of the synovial joints that causes chronic inflammation and leads to its progressive destruction [1,2]. It is more common in females than in males and has a peak incidence in patients aged 40–60 [3]. Typically, patients with RA experience pain and swelling in multiple joints bilaterally. The pain is accompanied by morning stiffness that can last over an hour [4]. In a clinical setting, the diagnosis is made based on the European League Against Rheumatism (EULAR) and the American College of Rheumatology (ACR) criteria [5]. With the rapid aging of the population, RA prevalence is increasing and constitutes a severe burden on health care systems worldwide.

Insights into the molecular pathways involved in the disease have led to the development of novel therapeutic strategies, such as biologic drugs and small-molecule inhibitors [6]. Despite those recent successes, a significant portion of patients still experience debilitating symptoms and suffer from progressive functional loss and disability. Therefore, further research into cellular and molecular mechanisms underlying synovial damage is needed.

**Figure 1:**
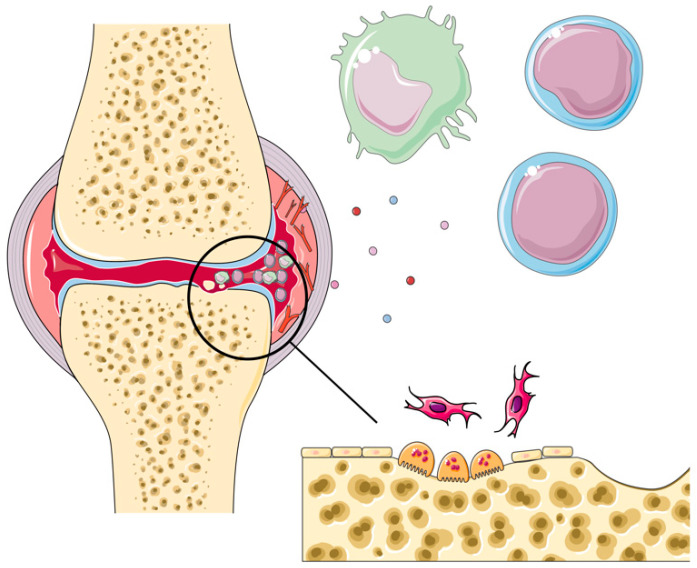
Graphical representation of the pathogenesis of rheumatoid arthritis. The hallmarks of RA, such as persistent inflammation, synovial hyperplasia, articular tissue degradation, and bony erosion, are the result of interactions between multiple cell types, such as antigen-presenting cells (APCs), mononuclear cells (T- and B-lymphocytes), fibroblast-like synoviocytes (FLSs), and chondrocytes.

Our current understanding of the pathogenesis of RA involves a combination of genetic, epigenetic, and environmental factors whose interplay results in synovial hyperplasia, inflammation, and progressive articular cartilage degradation with bony erosions (Figure 1) [7]. While genetic factors, such as genetic polymorphism, have been long established as risk factors of RA and elements of pathogenesis [8,9], recent studies have been investigating epigenetic mechanisms of gene expression regulation, such as microRNAs (miRs), as potential factors in the development of the disease.

The evidence suggests that miRs, through their ability to affect the expression of proteins involved in multiple molecular processes, are largely implicated in the pathogenesis of the disease [10,11]. Moreover, multiple studies have emerged, suggesting that their levels can serve as biomarkers to detect the presence of the disease before clinical symptoms arise and predict the severity of the disease course and response to treatment [12]. In this review we will focus on the studies that investigate the potential mechanisms through which miRs affect the disease process, and those that aim to identify select miRs as biomarkers of the disease. We searched the Medline database through PubMed and Google Scholar using the keywords “microRNA” and “rheumatoid arthritis”. Papers published from January 2005 to December 2024 were included, as well as related references of included papers. The language of the publications was restricted to English, while there was no limitation imposed on study types.

## 2. MicroRNAs

MiRs are short-chained non-coding RNAs that are posttranscriptional regulators of gene expression [13]. While miR sequences generally originate from the primary transcript within the target genes’ introns, they can also be located in the intergenic regions, distant from their target genes [11]. The process of miR biogenesis is a multi-step procedure that is initiated by transcription of miR by RNA Pol II (RNA Polymerase II) in the nucleus. Then, it either undergoes a canonical pathway that is dependent on Drosha processing or a noncanonical pathway where pre-miRs are generated from introns that undergo splicing and debranching [14,15].

Mature miRs possess the ability to bind to messenger RNAs (mRNAs) that have complementary sequences, thus halting the production of specific proteins. The efficacy of gene silencing is highly dependent on the level of complementarity to the target miRNA. If the match is sufficient, miRNAs induce 5′-to-3′ degradation of mRNA by enhancing its deadenylation and decapping [16]. In total, over half of protein-coding genes are regulated by miRs, in many cases by multiple species of miRs [17]. miRs have been identified to affect the release of pro-inflammatory cytokines and interferons [18] and are largely involved in regulating both T and B cells’ function, expansion, and differentiation [19,20]. Several miRs have been recognized as regulators of expression of genes and signaling pathways that are involved in the pathogenesis of rheumatic autoimmune diseases, such as systemic lupus erythematosus (SLE), ankylosing spondylitis (AS), or RA [21,22].

## 3. The Effect of miR in Synovial Cells on Key Cellular Processes Involved in the Pathogenesis of RA

The pathophysiology of RA is a complex process that involves the interaction of various immune cells, cytokines, and signaling pathways. The joint damage involves mainly the synovial membrane and is caused by the migration and activation of mononuclear cells (B cells, T cells, plasma cells, dendritic cells, and macrophages) [23]. In the early stages of the disease, the elements of innate immune response, such as dendritic cells, become activated by exogenous and autologous antigens. Antigen-presenting cells (APCs) then present those antigens to T cells, which mediate the inflammatory response [24]. The process of chronic inflammation causes the synovial membrane to become hyperplastic. Pannus, the portion of the synovial membrane abundant in osteoclasts, destroys the bone and the cartilage. This process is further affected by enzymes secreted by synoviocytes, chondrocytes, and neutrophils [23,24]. The degradation of cartilage in RA occurs when synoviocytes become activated by TNF-α (tumor necrosis factor alpha), IL-1 (interleukin 1), and Il-6 (interleukin 6), resulting in matrix metalloproteinase release. Additionally, chondrocytes also become activated by the cytokines, leading to further release of metalloproteinases.

**Figure 2:**
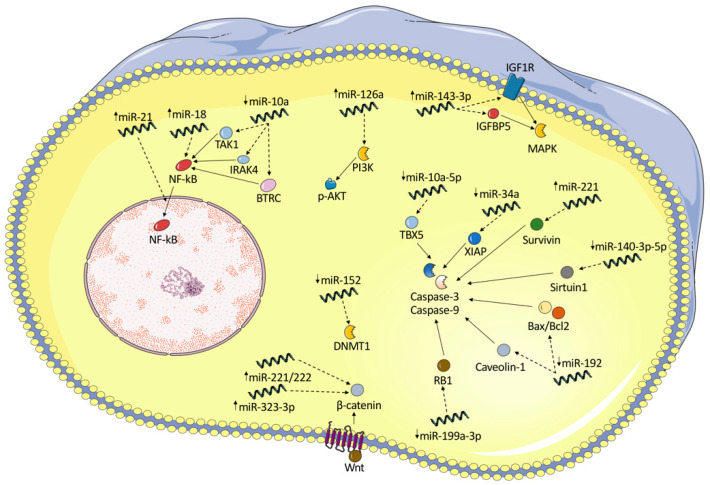
Graphical representation of the effect of miRNA on several key intracellular pathways in RA-FLSs, such as apoptosis, NF-κB, PI3K/p-AKT, MAPK, and Wnt/β-catenin pathways. Upregulation (arrow up) or downregulation (arrow down) of several miRNAs has been identified in the literature. Their indirect negative or positive effect on the expression of multiple target proteins is presented herein (dashed arrows). miRNA—microRNA; NF-κB—nuclear factor kappa-light-chain-enhancer of activated B cells; TAK1—TGF-beta-activated kinase 1; IRAK4—interleukin-1 receptor-associated kinase 4; BTRC—β-transducing repeat-containing protein 1; PI3K—phosphatidylinositol 3-kinase; p-AKT—phospho-protein kinase B; IGF1R—insulin-like growth factor 1 receptor; IGFBP5—insulin-like growth factor binding protein 5; MAPK—Ras/p38 mitogen-activated protein kinase; TBX5—T-box transcription factor 5; XIAP—X-linked inhibitor of apoptosis protein; RB1—retinoblastoma 1; DNMT1—DNA methyltransferase.

In this dissertation, we will focus on miRs that affect the proteins and pathways that result in the development and progression of RA regarding the pathways affected in different types of tissues—synoviocytes, fibroblast-like synoviocytes, osteoblasts, osteoclasts, and different types of peripheral blood mononuclear cells (PBMCs) (see Table 1 and Table 2). Notably, several key pathways and processes have been identified, including those involved in cell proliferation, cell death regulation, and many more (Figure 2).

### 3.1. Dysregulation of Molecular Pathways in Synoviocytes and Fibroblast-like Synoviocytes

Fibroblast-like synoviocytes (FLSs) and macrophages are key effector cells implicated in the pathology of RA. FLSs are cells of mesenchymal origin that, under physiological conditions, are components of the synovial lining of joints. Their role includes production of the extracellular matrix and synovial fluid to maintain the homeostasis of cartilage surfaces [66,67]. In RA, FLS increase in number and display a characteristic phenotype that includes dysregulated proliferation rate, reduced rate of apoptosis, increased migration and invasion abilities, and increased production of ECM (extracellular matrix) proteins and MMPs (matrix metalloproteinases) [68]. Researchers have identified that disturbed expression of multiple miRs, affecting various cellular processes, may lead to the presentation of this specific phenotype of FLS. Herein, we will summarize the findings of the studies with regard to the molecular pathway affected.

#### 3.1.1. NF-κB

The release of NF-κB (nuclear factor kappa-light-chain-enhancer of activated B cells) is triggered by pro-inflammatory cytokines such as IL-1 and TNF-α. Receptor activator of NF-κB (RANK) and its ligand (RANKL) are involved in the process [69]. Recent studies show the upregulation of RANKL in RA-FLS and activation of its downstream pathways involving NF-κB [70]. Several MiRs are responsible for regulating this pathway in FLS, thus contributing to the pathogenesis of RA. MiR-21 has been found to be overexpressed in patients suffering from RA. Its overexpression in FLS may promote cell proliferation by facilitating the nuclear translocation of NF-κB in an animal model of RA [37]. A relationship has been discovered where miR-17-92-derived miR-18a contributes to cartilage destruction through a positive feedback loop in NF-κB signaling in RA FLS obtained from RA patients. TNF-α induces the expression of miR-18a, which in turn targets NF-κB pathway inhibitor TNF-α-induced protein 3 [31]. Similarly, an NF-κB/YY1/miR-10a/NF-κB regulatory circuit exists that promotes the excessive secretion of NF-κB-mediated inflammatory cytokines and subsequent proliferation and migration of RA FLSs. miR-10a was found to be downregulated in FLSs of human RA patients, which could accelerate NF-κB activation by targeting interleukin-1 receptor-associated kinase 4 (IRAK4), TGF-beta-activated kinase 1 (TAK1), and β-transducing repeat-containing protein 1 (BTRC) [29].

#### 3.1.2. Apoptosis and Cell Cycle

Activated FLS in RA are characterized by their prolonged lifespan and acquired anti-apoptotic properties, both of which lead to further destruction of the cartilage [68]. Several miRs have been identified to regulate the process of apoptosis in RA-FLS. The downregulation of miR-10a-5p promotes cell proliferation and restricts apoptosis by targeting T-box transcription factor 5 (TBX5) in synoviocytes. Conversely, the overexpression of miR-10a-5p promotes forced cell death in an in vitro model [28]. Basal expression levels of miR-34a were reduced in RA patients, and its levels were independent of cytokine stimulation. The level of miR-34a was inversely correlated with the expression of X-linked inhibitor of apoptosis protein (XIAP), which was found to be a direct target of miR-34a. Moreover, the overexpression of miR-34a led to an increased rate of FasL (Fas ligand)- and TNF-related apoptosis-inducing ligand (TRAIL)-mediated apoptosis [38]. Furthermore, the expression of miR-143-3p was found to be elevated in synovium tissues of RA patients. Experimental data suggests that miR-143-3p regulates cell proliferation and apoptosis by targeting insulin-like growth factor 1 receptor (IGF1R) and insulin-like growth factor binding protein 5 (IGFBP5), thereby regulating the Ras/p38 mitogen-activated protein kinase (MAPK) signaling pathways [46]. miRs 140-3p and 140-5p are also downregulated in rheumatoid arthritis synovial fibroblasts (RASFs) of human RA patients. Experimental overexpression of miRs 140-3p and 140-5p in a rodent model reduced the expression of target molecules sirtuin 1 and stromal cell-derived factor 1. Moreover, the overexpression resulted in increased cell apoptosis and reduced proliferation and migration abilities [47]. miR-152 levels were decreased in serum, synovial tissue, and RA-FLS in comparison with healthy controls. On the contrary, the expression of miR-221 in synovial tissues of patients with RA was significantly higher than in healthy controls. Subsequently, the downregulation of miR-221 suppressed the expression of pro-inflammatory cytokines and chemokines and inhibited cell migration. Moreover, miR-221 decreased the expression of survivin and XIAP. This effect led to the induction of apoptosis in stimulated FLS cells [56]. Both RA synovial tissues and RA-FLSs had significantly lower levels of miR-192 when compared to healthy controls. The overexpression of miR-192 inhibited proliferation and caused a cell cycle arrest at the G0/G1 phase. Additionally, it resulted in apoptosis accompanied by an increase in Caspase-3 activity and Bax/Bcl-2 ratio. The data suggests that Caveolin 1 is a direct molecular target of miR-192 [52]. Similarly, the expression of miR-199a-3p was significantly reduced in RA-FLS. Its overexpression in vitro induced apoptosis and inhibited proliferation. miR-199a-3p directly targeted the Retinoblastoma 1 (RB1) gene [53]. miR-338-5p was significantly upregulated in RA-FLS and facilitated their proliferation rate, migration, and invasion. Nuclear factor of activated T cells 5 (NFAT5) was identified as a downstream target of miR-338-5p in FLS isolated from RA patients [58]. On the contrary, the levels of miR-124a were significantly decreased in human RA synoviocytes. Its upregulation in experimental conditions suppressed FLS’ proliferation, arresting the cell cycle at the G1 phase. miR-124a binds directly to the 3′-untranslated region of cyclin-dependent kinase 2 (CDK-2) and monocyte chemoattractant protein 1 (MCP-1) mRNA [39]. miR-137 has previously been shown to be involved in regulating cell proliferation, migration, and apoptosis in a variety of cells [71]. miR-137 was downregulated in RA-FLS in comparison with normal control FLS. Its overexpression in an animal model resulted in reduction in proliferation, migration, and invasion [45]. Luciferase reporter assays indicated that miR-137 targets C-X-C motif chemokine ligand 12 (CXCL12), which is involved in chronic inflammation [72] and whose expression is increased in inflammatory conditions, such as RA [73]. The Toll-like receptor 4 (TLR4)/NF-kB pathway might also affect FLS migration and invasion. miR-27a, which was significantly downregulated in both the serum and FLS of RA patients, is a key regulator of follistatin-like protein 1 (FSTL1) expression. In cultured FLS isolated from patients, miR-27a inhibition promoted further migration and invasion of FLS, while miR-27a upregulation resulted in inhibition of FSTL1 expression and inhibition of the TLR4/NF-κB pathway [36].

#### 3.1.3. PI3K/AKT Signaling Pathway

The phosphatidylinositol 3-kinase (PI3K)/protein kinase B (AKT) signaling pathway is involved in the pathogenesis of inflammation. Cytokines released from FLS lead to the activation of the PI3K/AKT signaling pathway by binding to specific receptors, promoting migration and invasion of those target cells [74]. Several miRs have been identified that affect the proliferation and apoptosis of RASFs by regulating the PI3K/AKT signaling pathway. Lentiviral transduction of miR-126 into RASFs in vitro promoted RASF proliferation and inhibited apoptosis. Levels of Phosphoinositide-3-Kinase Regulatory Subunit 2 (PIK3R2) were decreased, while the total PI3K and p-AKT levels were increased. Luciferase reporter assays showed that miR-126 directly targeted *PIK3R2*. The authors propose that inhibiting miR-126 may yield therapeutic benefit in the treatment of RA [75]. However, the findings of other studies indicate that miR-126 downregulation in RA FLS is associated with increased cytokine production. This discrepancy likely reflects compartment- or context-specific effects, and further studies are needed to better elucidate the role of miR-126 in RA.

#### 3.1.4. Cytokines

Cytokine and chemokine production is one of the main hallmarks of inflammation in RA. Several studies have investigated the regulatory role of miRs on the production of cytokines. The regulation can be both direct and indirect by influencing the expression of genes that regulate cytokine and enzyme production.

Toll-like receptor (TLR) family is a type of immune receptor that is expressed on the surface of immune cells and FLS [76]. Toll-like receptor 2 (TLR2) promotes inflammation by initiating the immune response and participating in the release of cytokines and MMPs [77]. It was found that TLR2 expression is upregulated in RA [78]. The expression of miR-19 was significantly downregulated in FLS from RA patients compared with controls. The TLR2 gene was identified as the target gene of miR-19. In a human study, upregulation of miR-19 resulted in inhibition of IL-6 and MMP-3 release in FLS [32]. The exact pathway through which miR-19 exerts its biological function is still unknown; some in vitro data suggest it might regulate suppressor of cytokine signaling 3 (SOCS3) expression to enhance the Janus kinase-signal transducer and activator of transcription (JAK-STAT) pathway [79]. Another study identified miR-19b to be downregulated in activated human cultured RA FLS and predicted it to directly target TLR2 mRNA, which regulates IL-6 and matrix metalloproteinase 3 release [33]. miR-20a, which belongs to the miR-17-92 cluster, has been identified as a negative regulator of inflammation in RA FLS. Stimulation of FLS induces a drop in expression of miR-20a, which modulates the expression of apoptosis signal-regulating kinase 1 (ASK1). ASK1 is a member of the TLR4 pathway, which is upstream of p38-MAPK.

Upregulation of miR-20a led to a global decrease in IL-6 secretion [25]. The role of IL-17 (Interleukin-17) has been well elucidated in many autoimmune inflammatory diseases as it largely contributes to both the initiation of chronic inflammation and autoimmunity [80]. The IL-17 cytokine family consists of six ligands (IL-17A-F) and five receptors (IL-17RA-E). Interleukin 23 (IL-23) mediates the proliferation of Th17 cells and the production of IL-17. Alongside TNF-α, it is one of the key components determining chronic inflammation in RA [81,82]. The expression of miR-126 was downregulated in human RA patients when compared with healthy controls, whereas the expression of TNF-α, interferon gamma (IFN-y), and interleukin 23 receptor (IL-23R) was upregulated. Furthermore, the overexpression of miR-126 inhibited the expression of IL-23R [42].

In a mouse model, the expression of miR-223-3p is upregulated in RA and results in suppression of IL-17 receptor D (IL-17RD) expression and may contribute to the pathogenesis of RA [57]. In inflammation that accompanies autoimmune diseases, IL-17 downregulates miR-23b expression in FLS in both humans and rodents. In turn, miR-23b suppresses IL-17 and other cytokines by targeting TGF-β-activated kinase 1/MAP3K7 binding protein 2 (TAB2), TGF-β–activated kinase 1/MAP3K7 binding protein 3 (TAB3), and inhibitor of nuclear factor κ-B kinase subunit α (IKK-α) and acts to suppress autoimmune inflammation. Therefore, IL-17 contributes to the pathogenesis of RA by suppressing the expression of anti-inflammatory miR-23b [27]. Monocyte chemoattractant protein 1 (MCP1) attracts memory T-lymphocytes and NK cells, which contribute to the pathogenesis of RA [83]. MCP1 is upregulated in RA patients and is involved in the pathogenesis of the disease [40]. Experimental data in human-derived tissue show that the expression of MCP1 is regulated by miR-124a, whose levels are significantly decreased in RA synoviocytes. miR-124 was identified to directly suppress cyclin-dependent kinase 2 (CDK2) and MCP1, thereby decreasing the chemotactic effects [40]. Another miR, miR-346, has been identified to be downregulated in human-derived RA FLS. miR-346 was demonstrated to indirectly inhibit Bruton’s tyrosine kinase (Btk) and control the synthesis of interleukin 18 (IL-18) by FLS. Therefore, its expression may be pivotal to prevent an excessive inflammatory response [59]. Several genes that affect B cell survival are also regulated by miRs. One of them, B cell-activating factor (BAFF), is a member of the TNF superfamily and is responsible for the survival and homeostasis of naïve and transitional B cells, plasmablasts, and plasma cells [84]. In RA, FLS produce substantial concentrations of BAFF, which enables them to recruit B cells [85]. The expression levels of miR-30a-3p were downregulated in human RA FLS. Moreover, upregulation of miR-30a-3p resulted in decrease in BAFF synthesis and release, thus decreasing B cell survival in an experimental model [26]. Additionally, stimulated human FLS secrete several autocrine/paracrine mediators, including Cysteine-rich angiogenic inducer 61 (Cyr61). Overexpressed Cyr61 acts to promote FLS proliferation and leads to synovial hyperplasia [86]. In human RA FLS, miR-22 was shown to directly target the 3′-UTR of Cyr61 messenger RNA, inhibiting its expression. In RA, mutant forms of p53 lose the ability to activate the expression of miR-22, leading to its decreased levels and overexpression of Cyr61 [35].

TNF-α is a pro-inflammatory cytokine that regulates the inflammatory response in RA. It is one of the most crucial cytokines that causes inflammation in the joint [87]. It is known to increase proliferation, cytokine production, and differentiation of immune cells and synovial cells, and promote angiogenesis [88,89]. Several studies have investigated the impact of changes in miR levels on expression of genes involved in TNF-α signaling. In an animal model of RA, miR-17 expression was significantly lower in serum, SFs, and synovial tissue. miR-17 induced the destabilization of TRAF2 and reduced its ability to associate with cellular inhibitor of apoptosis 2 (cIAP2), resulting in the downregulation of TNF-α-induced NF-κBp65, c-Jun, and signal transducer and activator of transcription 3 (STAT3) nuclear translocation and production of IL-6, IL-8, MMP-1, and MMP-13 in human RA SFs [30]. Therefore, miR-17 acts as a negative regulator of TNF-α signaling by modulating the processes of ubiquitination.

Global miR profiling study revealed that miR-143 and miR-145 were upregulated in RA-FLSs of human patients compared to osteoarthritis fibroblast-like synoviocytes (OA-FLSs) and were highly expressed in RA-FLSs, and this change in expression affected the TNF-α pathway. A prediction model identified IGFBP5 and semaphorin 3A (SEMA3A) as potential target genes of miR-143 and miR-145, respectively. IGFBP5 deficiency resulted in upregulation of NF-κB signaling in RA-FLSs that was induced by TNF-α, resulting in higher IL-6 production, suggesting that miR-143 renders RA-FLSs more sensitive to TNF-α stimulation. Moreover, miR-145 reduced SEMA3A expression, resulting in greater stimulation by vascular endothelial growth factor (VEGF165) [44].

#### 3.1.5. Wnt

Activation of Wnt/cadherin signaling in RA has been reported in multiple studies, and the upregulation of β-catenin has been linked with the activated SF phenotype in RA [90]. β-catenin is a component of E-cadherin complexes, which mediates cell-to-cell adhesion, and key mediator of the Wnt signaling pathway [91]. RA-FLS remain persistently activated, increasing their proliferation, growth, and invasion [92]. In a transgenic mouse model of RA, the expression of miR-221/222 and miR-323-3p was notably higher in synovial fibroblasts in comparison with controls. Pathway enrichment analysis indicated Wnt and cadherin signaling as the top pathways potentially affected by both miR-221/222 and miR-323-3p overexpression [49]. In another study, the expression of miR-152 was downregulated in RA model rats, while the overexpression of miR-152 inhibited the canonical Wnt signaling (β-catenin-dependent) through DNA methyltransferase (DNMT1) inhibition. The authors hypothesize that increased DNMT1 could activate the canonical Wnt signaling through secreted frizzled-related protein 1 (SFRP1) inhibition via the DNA hypermethylation in its promoter during the disease [50].

#### 3.1.6. Osteogenesis

RA-FLS contribute to the production of pro-inflammatory cytokines and enzymes that degrade the extracellular matrix, leading to gradual bone erosions [93]. The gene expression pattern of FLS is similar to that of mesenchymal stem cells, and FLS can differentiate into chondrocytes and osteoblasts [94]. Forced cell differentiation is speculated to be a candidate therapeutic option for RA. After osteogenic differentiation, 12 miRNAs were upregulated, and 24 miRNAs were downregulated in a miRNA array study in cultured RA-FLS isolated from human tissue [54]. miR-218 was identified as a crucial inducer of the osteogenic differentiation of RA-FLS. MiR-218 modulates the osteogenic differentiation of RA-FLS through the roundabout 1 (ROBO1)/dickkopf-1 (DKK-1) axis. Therefore, the induction of the osteogenic differentiation of proliferating RA-FLS might become a therapeutic strategy for RA [54]. Another study, in a mouse model, identified up- and downregulated miRNAs that both inhibit and promote bone formation. These miRNAs were predicted to target Wnt and bone morphogenetic protein (BMP) signaling pathways [55]. Moreover, the secretion of miR-221-3p was upregulated by SF when treated with TNF. It resulted in suppression of osteoblast differentiation and mineralization, suggesting that miRs might regulate the processes that affect bone loss and compensatory bone formation [55].

### 3.2. Dysregulation of miRs in PBMCs of RA Patients

A wide array of immune cells, both those constituting the innate and the adaptive immune systems, are implicated in initiating and mediating the autoimmune response in RA. Among those are CD4 T-lymphocytes, Treg cells, B cells, and monocyte–macrophage lineage cells. Numerous miRs have been investigated specifically in the context of their expression in PBMCs of human patients. Most studies found a correlation between the level of miR and the disease, but did not elucidate a potential molecular pathway through which the miRs can affect the predisposition to the disease [61,95,96,97]. Of the studies that did elucidate the exact mechanisms, the alterations mainly affect pathways related to cytokine secretion and Treg balance in the peripheral blood. In ACPA (anti-citrullinated protein antibody)-positive patients specifically, ACPA leads to a decreased expression of let-7a in monocytes. The decrease in let-7a levels facilitates inflammation by ACPA-mediated phosphorylation of extracellular signal-regulated kinase 1/2 (ERK1/2) and c-Jun N-terminal kinase (JNK) that leads to increased expression of pro-inflammatory IL-1β [60]. Interestingly, in another study [61], let-7a expression did not significantly differ in patients with RA and healthy controls. In this study, the expression of miR-146a, miR-155, miR-132, and miR-16 was elevated in RA PBMC when compared to healthy controls. Moreover, despite increased expression of miR-146a in patients, the expression of its two molecular targets, tumor necrosis factor receptor-associated factor 6 (TRAF6) and IL-1 receptor-associated kinase 1 (IRAK-1), was unchanged [61]. Furthermore, the expression of miR-155 inversely correlated with the expression of pro-inflammatory cytokines, namely TNF-α and IL-1B. The study suggests that miR-155 targets and suppresses the expression of suppressor of cytokine signaling 1 (SOCS1) gene, leading to cytokine upregulation [62]. However, another study found that miR-155 is upregulated in both PBMCs and RA-FLS and may be protective against inflammation by attenuating the expression of IKBKE (IκB kinase-ε complex), which is a known protein whose expression contributes to joint destruction in RA [63,98]. In recent years, several studies have identified the dual role of miR-155 in the pathogenesis of RA. On one hand, the upregulation of miR-155 in RA FLS is assumed to be protective against the acquisition of a destructive phenotype by RA FLS by means of repressing the expression of matrix metalloproteinases by targeting IKBKE. However, miR-155 plays a significant pro-inflammatory role as it participates in monocyte recruitment, augmenting chemokine production, and facilitates Th1 immune response. While current data is highly suggestive of the diagnostic and therapeutic potential of miR-155, further studies are needed to gain a better understanding of the exact role of miR-155 in RA pathogenesis. It is important to acknowledge these compartment-specific effects of miR-155. Data suggests that while miR-155 inhibition reduces disease severity in an animal model of RA, a certain level of endogenous miR-155 expression is required for normal immune system function.

The expression of miR-548a-3p in both PBMCs and serum exosomes was downregulated in RA patients and was negatively associated with parameters of disease activity. In a cell culture model, miR-548-3p inhibited the activation of macrophage-like cells by regulating the TLR4/NF-κB signaling pathway [99]. Some of the miRs that affect the Th17/Treg balance include miR-301a-3p. This miR is overexpressed in PBMCs of RA patients and correlates with frequency of Th17 lymphocytes. miR-301a-3p inhibits PIAS3 (protein inhibitor of activated STAT3), which in turn upregulates STAT3, a transcription factor of Th17 cells [64]. Moreover, a reverse correlation between miR-21 and STAT3 expression was found, where decreased levels of miR-21 in PBMCs of RA patients were accompanied by changes in STAT3 expression and activation. A feedback loop is suspected, whose dysregulation may contribute to the Th17/Treg imbalance [65].

## 4. Therapeutic Potential of miRNA in the Treatment of RA

Currently established first-line medications for RA patients include disease-modifying anti-rheumatic drugs (DMARDs), primarily methotrexate (MTX), which aim to achieve full clinical remission or low disease activity within 6 months of treatment. While the addition of several new-generation small molecule inhibitors and biologic agents has led to an increased rate of disease management, they produce only partial response rates in some patients and are accompanied by severe long-term side effects. Therefore, the search for new and efficacious therapies is warranted.

Recent findings indicate that several miRNAs can be a potential target of novel therapies in RA treatments (see Table 3). However, although multiple studies have identified multiple miRNAs that are differently expressed in RA, multiple obstacles must be overcome before translating those findings to therapeutic success. Several molecular mechanisms through which the destruction of pannus and bone could be halted have been identified in in vitro studies. Modulation of the Wnt and NF-κB pathways, as well as apoptosis and cell cycle, could become a successful therapeutic method. In an in vitro model, administration of miR-124a decreased the proliferation of RASF by arresting the cell cycle and interfered with chemotaxis of inflammatory cells by suppressing the production of cyclin-dependent kinase 2 (CDK-2) and monocyte chemoattractant protein (MCP-1) [39]. Moreover, miR-26b mimic increased apoptosis and diminished RASF proliferation [100] via the Wnt/Glycogen synthase kinase-3 beta (GSK-3β)/beta-catenin pathway.

Studies on animal models have produced further evidence of the potential efficacy of miRNA therapy in RA. The goal of these therapies is to alter the levels of target miR activity to regulate the expression of its target gene. Two therapeutic strategies currently involve the use of inhibitors and mimics or miRs. Mostly, chemically modified miR agomirs and antagomirs are used in vivo. Moreover, two methods of administration are routinely performed—systemic or local administration.

Intravenous treatment with chemically modified miR-34a inhibitor ameliorated murine arthritis, downregulated T cell percentage and cytokine expression, and suppressed bone loss [101]. However, the exact molecular targets of miR-34a have not been determined in vivo. Similarly, intravenous injection of miR-708-5p mimics reversed the activation of the Wnt pathway and could ameliorate RA symptoms, such as synovial hyperplasia and joint destruction [102]. However, systemic administration of miRs possesses several drawbacks, one of which is their unfavorable biodistribution leading to tissue accumulation and rapid renal clearance [103]. Therefore, intra-articular administration was the preferred method of administration in several studies. Intra-articular administration of miR-15 resulted in increased apoptosis of synovial cells as measured by caspase-3 expression. Bcl-2, an apoptosis inhibitor, has been identified as a target of miR-15 [104]. Intra-articular injection of miR-141-3p mimic or siRNA targeting forkhead box protein C1 (FoxC1) resulted in silencing of the FoxC1/B-catenin axis, which led to decreased cartilage destruction [105]. The results of these studies, as well as many others, can be found in Table 4.

**Table 3:** Summarizes the findings investigating miRs as diagnostic tools and predictors of disease course in rheumatoid arthritis. Abbreviations (in alphabetical order): RA—rheumatoid arthritis; ERA—early rheumatoid arthritis; HC—healthy controls; OA—osteoarthritis; SLE—systemic lupus erythematosus.

miRNA	Effect	Comment	Reference
miR-106a-5p, miR-148b-3p and miR-199a-5p, miR-143-5p and miR-346	Serum levels of miR-106a-5p, miR-148b-3p and miR-199a-5p were decreased whereas serum miR-143-5p and miR-346 levels were increased in patients with RA compared to healthy individuals	RA vs. healthy individuals	[106]
miR-223	miR-223 discriminated RA patients from controls with AUC = 0.85	RA vs. healthy individuals	[107]
miR-146a, miR-155 and miR-16	miR-223 in treatment naïve ERA correlated with disease activity, while miR-16 and miR-223 are possible predictors for disease outcome in ERA (correlated with DAS28)	Early RA vs. late RA. neither miR-16 nor miR-223 could distinguish ERA from HC	[102]
miR-22	miR-22 is associated with progression from systemic autoimmunity to RA inflammation	Pre-clinical vs. early-stage RA	[108]
miR-126-3p, let-7d-5p, miR-431-3p, miR-221-3p, miR-24-3p, miR-130a-3p, miR-339-5p, let-7i-5p, and miR-17-5p	miR-126-3p, let-7d-5p, miR-431-3p, miR-221-3p, miR-24-3p, miR-130a-3p, miR-339-5p, let-7i-5p were significantly elevated in RA serum compared to HC (all *p* < 0.01) and 1 miRNA (miR-17-5p) was significantly lower in RA	RA patients vs. healthy controls	[109]
miR-103a-3p, miR-155, miR-146a-5p, miR-26b-3p, miR-346	miR-103a-3p, miR-155, miR-146a-5p, and miR-26b-3p was significantly upregulated, whereas miR-346 was significantly downregulated in RA patients in comparison with healthy controls	RA patients, first-degree relatives, and healthy controls	[84]
miR-15a-5p, miR-24-3p, miR-26a-5p, miR-125a-5p, miR-146a-5p, miR-155-5p, and miR-223-3p	miR-15a-5p, miR-24-3p, miR-26a-5p, miR-125a-5p, miR-146a-5p, miR-155-5p, and miR-223-3p were significantly increased in patients with RA. The highest accuracy for diagnosis of RA was identified for the combination of miR-24-3p, miR-26a-5p, and miR-125a-5p	RA vs. healthy controls	[103]
miR-223-3p, miR-16-5p	miR-223-3p and miR-16-5p, were significantly lower in the sera from early RA patients than in those from established RA patients and healthy controls. miR-16-5p was higher in patients with established RA than in healthy control samples	Early RA vs. long-standing RA vs. healthy controls	[110]
miR-4634, miR-181d, miR-4764-5p, miR-342-3p, miR-3926, miR-3925-3p, miR-122-3p, miR-9-5p and miR-219-2-3p	miR-4634, miR-181d and miR-4764-5p expression levels were increased, whereas miR-342-3p, miR-3926, miR-3925-3p, miR-122-3p, miR-9-5p and miR-219-2-3p expression levels were decreased in RA patients vs. controls. miR-4764-5p, miR-4634, miR-9-5p and miR-219-2-3p exhibited significant correlations with either plasma cytokine and chemokine levels or clinical features	RA vs. healthy controls	[111]
miR-24, miR-125a-5p	miR-24, miR-125a-5p, were higher in patients with RA, ACPA-negative	RA vs. healthy controls vs. OA/SLE	[112]
miR-132, miR-16, miR-146a	miR-132 was higher in HCs and differentiated them from patients with RA or OA. miR-16 and miR-146a concentration correlated with disease activity	RA vs. OA vs. healthy controls	[113]
miR-210, miR-155	MiR-210 was lower in RA compared to controls correlated inversely with disease activity and laboratory variables. MiR-155 expression was increased in RA compared and correlated with laboratory values of cytokines	RA vs. healthy controls	[12]

**Table 4:** Summarizes the findings of studies investigating the potential therapeutic effect of miRNA modulation in an animal model. Abbreviation: Bcl-2—B cell lymphoma 2.

Target	Agent (Mimic/Inhibitor)	Model	Delivery System & Route/Dose	Efficacy	Safety	Reference
miR-15a	Mimic	Auto-antibody arthritis mice	Double-stranded miR-15a-atellocollagen complex intraarticular injection	Induction of apoptosis by silencing the expression of Bcl-2	Potential off-target risk in the liver	[104]
miR-140-3p	Mimic	Collagen-induced arthritis and collagen antibody-induced arthritis mice	Lentiviral-mediated transfection of pre-miR-140 precursor molecules	Increased cell apoptosis, reduced proliferation and migration rate and production of cytokines through reduction of Sirtuin 1 expression. Lower clinical arthritis scores.	Late injections fail to achieve therapeutic responses	[47]
miR-140-5p	Mimic	Collagen-induced arthritis and collagen antibody-induced arthritis mice	Lentiviral-mediated transfection of pre-miR-140 precursor molecules	Increased cell apoptosis, reduced proliferation and migration rate and production of cytokines. Lower clinical arthritis scores.	Late injections fail to achieve therapeutic responses	[47]
miR-150-5p exosomes	Mimic	Collagen-induced arthritis mice	Mesenchymal stem cell -derived miR-150-5p exosomes	Decreased migration and invasion in RA FLS. Reduced joint destruction by inhibiting synoviocyte hyperplasia and angiogenesis. Lower clinical arthritis scores.	No safety profile data	[114]
miR-26a	Mimic	Pristane induced arthritis rats	Intraperitoneal administration of miR-26a mimic	Results in decreased cytokine expression and ameliorates the disease severity assessed by arthritis severity scores	Potential off-target risk in the spleen	[115]
miR-223	Inhibitor	Collagen-induced arthritis mice	Intraperitoneal injection of lentiviral vectors expressing miR-223 target sequence	Treatment reduced the arthritis score and less severe bone erosion in histopathologic slides	Widespread biodistribution of the vector. No histopathological assessment of off-target activity was performed	[116]
miR-106b	Inhibitor	Collagen-induced arthritis mice	Orbital injection with lentiviral-mediated miR-106b inhibitor	decreased arthritis incidence and attenuated bone destruction and histological severity	No safety profile data	[117]
miR-34a	Inhibitor	Collagen-induced arthritis mice	Intravenous treatment with chemically modified miR-34a inhibitor	Decreased arthritis scores, alleviation of joint swelling, suppressed bone loss	Low tissue selectivity of miR-34a	[101]
miR-708-5p	Mimic	Collagen-induced arthritis rat	Intravenous injection of miR-708-5p mimics	Restoration of miR-708-5p levels improves arthritis index	No safety profile data	[102]
miR-146a	Mimic	Collagen-induced arthritis mice	Intravenous injection of miR-146a	Administration of miR-146a partly prevented joint destruction (histological evidence). No differences in joint swelling.	Potential for off-target adverse effects in liver, spleen, and kidney	[118]
miR-141-3p	Mimic	Collagen-induced arthritis rats	Intra-articular injection of miR-141-3p mimic	miR-141-3p injection results in lower arthritis scores and reduced ankle joint swelling	miR-141-3p was expressed in target tissue 7 days post-injection.	[105]
miR-124	Mimic	Adjuvant-induced arthritis rats	Intra-articular injection of precursor miR-124	miR-124 suppressed arthritis, as demonstrated by decreased arthritis score and histopathological assessment	Evidence of systemic distribution	[119]

Another therapeutic strategy is the replacement of miR using lentiviral vectors. Lentiviral miR-140-3p and miR-140-5p transfection increased cell apoptosis, reduced proliferation and migration rates and production of cytokines, by targeting sirtuin 1 and stromal cell-derived factor 1, respectively [47]. Intraperitoneal injection of lentiviral vectors expressing miR-223 target sequence results in decreased miR-223 expression and an increase in nuclear factor 1A (NF-1A). The treatment reduced the arthritis score and bone erosions in mice [47]. However, lentiviral delivery of miR is associated with several side effects, including off-target effects or response of the innate immune system [47]. Therefore, future research must focus not only on mechanistic studies to investigate the molecular pathways affected by miRs, but also on finding solutions to safely apply those therapies in the clinic.

There have been several other strategies aiming to establish optimal miRNA delivery vessels that would allow for both tissue-specific targeting and adequate biostability while trying to minimize off-target effects and toxicity. One of the approaches involves using exosomes as delivery vessels for miRs. Several in vivo animal studies have been performed. In CIA mice, intraperitoneal administration of mesenchymal stem cell-derived miR-150-5p exosomes decreased migration and invasion of RA FLS and reduced clinical arthritis scores by targeting MMP14 and VEGF. Moreover, in vitro data suggest that bone marrow mesenchymal stem cell-derived miR-223 has been found to exert anti-inflammatory properties. These findings, however, have not been validated in an in vivo setting. In all, experimental data thus far is limited, and further studies are required to optimize methods for exosome isolation and address their poor biodistribution in vivo. There have been several attempts to augment the stability and bioactivity of exosomal surfaces by methods of bioengineering.

Aside from exosomes, miR-loaded nanoparticles have been investigated in the context of RA therapy. Inflammation-instructed nanoparticle delivery of IL-4/miR-21 has been found to attenuate inflammation via NF-κB inhibition in mice. Another potential therapeutic option that could enhance tissue delivery is ligand conjugation, a method in which MiR molecules are directly conjugated to a ligand. The ligand then binds to a surface receptor that is abundant on the target cells. However, more research is needed to determine the feasibility of this method in RA.

## 5. miRs as Diagnostic Tools and Predictors of Disease Course

Early introduction of treatment is key to stopping and preventing the progression of tissue destruction and preventing disability in patients with RA. While in most cases, the diagnosis is made mainly on the basis of medical history, clinical manifestation, and presence of autoantibodies, the introduction of characteristic miR expression profiles in patients might aid the early diagnosis of the disease, especially in the case of seronegative form of the disease. Circulating miRs are a promising biomarker for application in clinical practice as they can easily be detected and quantified by widely available laboratory techniques.

The results of multiple human studies indicate that levels of circulating miRs differ in RA patients in comparison with healthy controls. However, the clinical utility of assessing levels of single miRs is rather limited, as in some cases the miRs fail to distinguish RA patients from healthy subjects or the results of studies are conflicting [120,121,122]. To achieve the strongest diagnostic accuracy, the studies investigated panels of miRs to identify the most sensitive and specific combinations of miRs. A combination of increased miR-24, miR-26a, and miR-125a achieved the highest diagnostic accuracy for RA (AUROC = 0.747) [122], regardless of RF and ACPA status. In seropositive RA, increased levels of miR-26b, miR-103a, miR-146, and miR-155 were found, along with reduced levels of miR-346 [95]. Moreover, miR-21 was lower in RA compared to controls, and miR-155 was increased compared to controls, and both correlated with disease activity [12].

## 6. Conclusions

As presented by the recent evidence, miRs play a significant role in the development of RA. The dysregulation of gene expression by miRs is undoubtedly one of the driving factors in the pathogenesis of the disease and can affect multiple pathways and cellular processes, including cell differentiation, proliferation, and functioning of multiple cell types in different tissues. However, the interplay between miR and other epigenetic factors remains largely unexplored. While the studies have identified multiple miRs that affect genes in FLSs, immune cells, or osteocytes, there exists a lack of mechanistic evidence underlying those processes. In the future, further in vivo experiments should be performed to investigate the role of miRs in RA. Utilizing animal models of arthritis, gene knockout studies should be performed to provide further evidence of the function of the specific miRs. Currently, the evidence is rather limited, as the majority of the studies are performed in vitro, often providing only a correlation between the levels of miRs and the occurrence of RA without exploring the molecular basis of those relationships.

The clinical potential of miR as biomarkers of early disease that would allow for early diagnosis is significant. Circulating miRs have been proposed as novel biomarkers that would allow clinicians to diagnose the disease, monitor its progression, and assess response to treatment. However, this field remains largely unexplored, with more large-scale studies needed to verify the utility of circulating miR in clinical practice. Thus far, several miRs have been identified as predictive and prognostic markers in RA, but further studies are necessary. While several studies have identified different serum miR expression profiles compared to healthy controls, the ability to distinguish RA and OA is currently rather limited. In order to translate the findings into clinical practice, miR profiling needs to be able to precisely identify RA patients among other inflammatory autoimmune disorders. Moreover, those miR changes should be present at a very early stage so that they would meaningfully translate to improved clinical outcomes. Additionally, recent studies show that miR profiling has the potential to be used to predict response to conventional treatment.

Understanding the molecular mechanisms of miR deregulation in RA would allow for introduction of miR-based therapies in RA. The ability to up- or downregulate specific pathways through miR therapies holds significant potential. However, those therapies are still in their infancy, as the evidence is rather limited. Moreover, the therapies are associated with a significant risk of off-target effects. Therefore, more in vivo animal studies are needed before those therapies can be tested in a clinical setting. The evidence suggests that systemic administration of miRs results in unsatisfactory biodistribution, quick renal clearance, and accumulation in the liver. Recently, in order to improve tissue specificity and bioavailability, several new methods of miR delivery have been explored. While lentiviral vectors deliver miR effectively, multiple concerns have been raised regarding off-target effects. Other delivery systems show promise in pre-clinical studies, including miR-loaded nanoparticles and miRs conjugated to a ligand. While early results have been promising, more studies are needed before any of those therapies can be utilized in a clinical setting.

## Figures and Tables

**Table 1:** Summarizes the findings of studies investigating the expression of select miRNAs in synovial tissue. Abbreviations (in alphabetical order): ASK1—Apoptosis Signal-Regulating Kinase 1; BAFF—B cell-Activating Factor; Bax/Bcl-2—Bcl-2-Associated X Protein/B cell Lymphoma 2; BMP—Bone Morphogenetic Protein; BTRC—Beta-Transducin Repeat-Containing E3 Ubiquitin Protein Ligase; BTK—Bruton’s Tyrosine Kinase; CAV1—Caveolin-1; CDK-2—Cyclin-Dependent Kinase 2; cIAP1—Cellular Inhibitor of Apoptosis Protein 1; cIAP2—Cellular Inhibitor of Apoptosis Protein 2; Cyr61—Cysteine-Rich Angiogenic Inducer 61; DNMT1—DNA (Cytosine-5)-Methyltransferase 1; FLS—Fibroblast-like Synoviocytes; FSTL1—Follistatin-Like 1; IFN—Interferon; IGF1R—Insulin-Like Growth Factor 1 Receptor; IGFBP5—Insulin-Like Growth Factor Binding Protein 5; IL-1β—Interleukin-1 Beta; IL-17—Interleukin-17; IL-17RD—Interleukin-17 Receptor D; IL-23R—Interleukin-23 Receptor; IL-6—Interleukin-6; IRAK4—Interleukin-1 Receptor-Associated Kinase 4; Itgβ1—Integrin Beta 1; MAPK—Mitogen-Activated Protein Kinase; MCP-1—Monocyte Chemoattractant Protein-1; MMP-1—Matrix Metalloproteinase-1; MMP-3—Matrix Metalloproteinase-3; MMP-9—Matrix Metalloproteinase-9; NF-κB—Nuclear Factor Kappa-Light-Chain-Enhancer of Activated B Cells; NFAT5—Nuclear Factor of Activated T Cells 5; NLRP3—NOD-, LRR-, and Pyrin Domain-Containing Protein 3; PI3K/AKT—Phosphoinositide 3-Kinase/Protein Kinase B Pathway; PIK3R2—Phosphoinositide-3-Kinase Regulatory Subunit 2; PSMD13—Proteasome 26S Non-ATPase Subunit 13; RB1—Retinoblastoma Protein 1; Ras/p38—Ras Protein/p38 Mitogen-Activated Protein Kinase Pathway; ROBO1—Roundabout Guidance Receptor 1; SCDF1—stromal cell-derived factor 1; SEMA3A—Semaphorin-3A; SFRP1—Secreted Frizzled-Related Protein 1; SFRP4—Secreted Frizzled-Related Protein 4; TAK1—Transforming Growth Factor Beta-Activated Kinase 1; TBX-5—T-box Transcription Factor 5; TLR2—Toll-Like Receptor 2; TNF—Tumor Necrosis Factor; TNFAIP-3—Tumor Necrosis Factor Alpha-Induced Protein 3; TRAF2—TNF Receptor-Associated Factor 2; TRAIL—TNF-Related Apoptosis-Inducing Ligand; USP2—Ubiquitin-Specific Peptidase 2; VEGF165—Vascular Endothelial Growth Factor 165 Isoform; WNT—Wingless/Integrated (Wnt Signaling Pathway); XIAP—X-Linked Inhibitor of Apoptosis Protein.

miRNA	Up/Down	Evidence Type	Compartment	Affected Molecular Pathway	Effect	Reference
miR-20a	Down	In vitro	FLS	ASK1/IL-6	Associated with overexpression of ASK1 and subsequent release of pro-inflammatory cytokines (IL-1, IL-6)	[25]
miR-30a-3p	Down	In vitro	Fibroblasts	BAFF	Strong decrease in BAFF synthesis and release, and thus B cell survival	[26]
miR-23b	Down	In vitro and animal model	FLS	IL-17	Under normal conditions, miR-23b represses autoimmune inflammation. IL-17 downregulates miR-23b	[27]
miR-10a-5p	Down	In vitro	Synoviocytes	TBX-5	Promotes proliferation and restricts apoptosis	[28]
miR-10a	Down	Human	FLS	NF-κB activation by targeting IRAK4, TAK1, and BTRC	promotes the excessive secretion of NF-κB-mediated inflammatory cytokines and the proliferation and migration of RA FLSs	[29]
miR-17	Down	Animal	FLS	TRAF2, cIAP1, cIAP2, USP2, and PSMD13	miR-17 acts as a negative regulator of TNF-α signaling	[30]
miR-18a	Up	Human	Synovial fibroblasts	TNFAIP-3	Positive feedback loop in NF-κB signaling	[31]
miR-19	Down	Human	FLS	TLR2	increased TLR2 expression and enhanced cytokine release	[32]
miR-19b	Down	Human and in vitro	FLS	TLR2	Controls TLR2 expression, miR-19b can act as negative regulator of inflammation	[33]
miR-20a	Down	In vitro	FLS	NLPR3	Downregulation of miR-20a increases the expression of NLRP3-inflammasome and the secretion of IL-1β and MMP-1	[34]
miR-22	Down	Human	FLS	Cyr61	P53 mediates overexpression of Cyr61 via miR-22, leading to joint inflammation	[35]
miR-27a	Down	Human	FLS	FSTL1	Downregulation of miR-27a promotes cell migration and invasion of RA-FLS by targeting FSTL1 and upregulating the TLR4/NFκB pathway	[36]
miR-21	Up	Animal	FLS	NF-κB	miR-21 overexpression results in increased NF-κB levels and cell proliferation rates	[37]
miR-34a	Down	Human	Synovial fibroblasts	XIAP	Decreased rate of FasL- and TRAIL-mediated apoptosis	[38]
miR-124a	Down	Human	Synoviocytes	CDK-2, MCP-1	Induces cell proliferation through overexpression of CDK-2 and MCP-1 proteins	[39]
miR-124a	Down	Human	Synoviocytes	CDK-2, MCP-1	Overexpression of CDK-2 and MCP-1 proteins	[40]
miR-124a	Down	In vitro	FLS	Itgβ1	TNF-α-stimulated cell proliferation and activation of the Ras-Erk1/2 pathway	[41]
miR-126	Down	Animal	FLS	TNF, IFN, IL-23R	TNF-α and IFN-γ production and IL-23R expression were significantly upregulated	[42]
miR-126	Up	Human	RASF	PIK3R2	Stimulation of the PI3K/AKT pathway, reduced apoptosis	[43]
miR-143	Up	Human	FLS	IGFBP5	Renders synoviocytes susceptible to TNF-α and VEGF165 stimuli	[44]
miR-145	Up	Human	FLS	SEMA3A	Renders synoviocytes susceptible to TNF-α and VEGF165 stimuli	[44]
miR-137	Down	Animal	FLS	CXCL12	Increased proliferation, migration, and invasion, and expression of inflammatory cytokines	[45]
miR-143-3p	Up	Human and in vitro	Synovium	IGF1R, IGFBP5	Regulates cell proliferation and apoptosis by regulating the Ras/p38 MAPK signaling	[46]
miR-140	Down	Human and animal	Synovial fibroblasts	Sirtuin1, SCDF1	Decreased apoptosis, increased proliferation, and cell migration	[47]
miR-146a	Up	Human	Synovial tissue	Unknown	Unknown	[48]
miR-323-3p	Up	Animal	Synovial fibroblasts	Wnt/cadherin	enhances Wnt pathway activation and decreases the levels of its predicted target	[49]
miR-152	Down	Animal	FLS	DNMT1, SFRP1, SFRP4	Promotes the canonical Wnt signaling	[50,51]
miR-192	Down	Human and in vitro	FLS	CAV1	Increases cell proliferation and decreases apoptosis—decrease in caspase-3 activity and altered Bax/Bcl-2 ratio	[52]
miR-199a-3p	Down	In vitro	FLS	RB1	Attenuation of apoptosis, decrease in caspase-3 activity and Bax/Bcl-2 ratio	[53]
miR-218-5p	Up/down	Human	FLS	ROBO1	Promotes osteogenic differentiation of FLS	[54]
miR-221	Up	Animal	Mouse synovial samples	Wnt, BMP	Promotes compensatory bone formation	[55]
miR-221	Up	Human	FLS	MMP-3, MMP-9, survivin, XIAP	Promotes the expression of pro-inflammatory cytokines and chemokines, increases FLS cell migration and invasion. Inhibits apoptosis	[56]
miR-223-3p	Up	Animal	Synovial cells	IL-17RD	downregulates the expression of IL-17RD and upregulates that of IL-6 in synovial cells	[57]
miR-338-5p	Up	Human	RAFLS	NFAT5	promotes RAFLS’s viability and proliferation, migration by targeting NFAT5	[58]
miR-346	Up	Human	FLS	BTK	Modulates the inflammatory response	[59]

**Table 2:** Summarizes the findings of studies investigating the expression of select miRNAs in peripheral blood mononuclear cells of rheumatoid arthritis patients. Abbreviations (in alphabetical order): ERK1/2—Extracellular Signal-Regulated Kinases 1 and 2; Foxp3—Forkhead Box P3; IRAK1—IL-1 Receptor-Associated Kinase 1; IKBKE—inhibitor of nuclear factor kappa-B kinase subunit epsilon; IL-1—Interleukin-1; IL-1β—Interleukin-1 Beta; JNK—c-Jun N-terminal Kinase; Let-7a—Lethal-7a microRNA; PBMCs—peripheral blood mononuclear cells; PIAS3—Protein Inhibitor of Activated STAT3; STAT3—Signal Transducer and Activator of Transcription 3; STAT5—Signal Transducer and Activator of Transcription 5; SOCS1—Suppressor of Cytokine Signaling 1; TRAF6—TNF Receptor-Associated Factor 6; TNF—tumor necrosis factor.

miRNA	Up/Down	Evidence Type	Compartment	Pathway	Effect	Ref.
Let-7a	Down	Human	PBMCs	JNK, ERK1/2	Increased expression of IL-1β	[60]
miR-146a	Up	Human	PBMCs	TRAF6, IRAK1	Production of pro-inflammatory cytokines—IL-1 and TNF-α	[61]
miR-155	Up	Human	PBMCs	Unknown	Production of pro-inflammatory cytokines—IL-1 and TNF-α	[61]
miR-132,	Up	Human	PBMCs	Unknown	Production of pro-inflammatory cytokines—IL-1 and TNF-α	[61]
miR-16	Up	Human	PBMCs	Unknown	Production of pro-inflammatory cytokines—IL-1 and TNF-α	[61]
miR-155	Up	Human	PBMCs	SOCS1	Increased cytokine production	[62]
miR-155	Up	Human	PBMCs	IKBKE	Attenuates inflammation	[63]
miR-301a-3p	Up	Human	PBMCs	PIAS3	Positively correlates with Th17 frequency	[64]
miR-21	Down	Human	PBMCs	STAT3, STAT5, Foxp3	Dysregulation of Th17/Treg balance through feedback loop	[65]

## Data Availability

No new data were created or analyzed in this study. Data sharing is not applicable to this article.
